# Comparison of UV, Peracetic Acid and Sodium Hypochlorite Treatment in the Disinfection of Urban Wastewater

**DOI:** 10.3390/pathogens10020182

**Published:** 2021-02-09

**Authors:** Silvia Bonetta, Cristina Pignata, Sara Bonetta, Giulia Amagliani, Giorgio Brandi, Giorgio Gilli, Elisabetta Carraro

**Affiliations:** 1 Dipartimento di Scienze della Vita e Biologia dei Sistemi, Università di Torino, 10123 Torino, Italy; 2 Dipartimento di Scienze della Sanità Pubblica e Pediatriche, Università di Torino, 10126 Torino, Italy; cristina.pignata@unito.it (C.P.); sara.bonetta@unito.it (S.B.); giorgio.gilli@unito.it (G.G.); elisabetta.carraro@unito.it (E.C.); 3 Dipartimento di Scienze Biomolecolari, Università degli Studi di Urbino “Carlo Bo”, 61029 Urbino, Italy; giulia.amagliani@uniurb.it (G.A.); giorgio.brandi@uniurb.it (G.B.)

**Keywords:** wastewater, disinfection, *Campylobacter*, *Salmonella* spp., *E. coli* O157:H7, STEC, indicator microrganisms, UV, peracetic acid, sodium hypochlorite

## Abstract

One source of water contamination is the release of wastewater that has not undergone efficient treatment. The aim of this study was to evaluate the reduction obtained with sodium hypochlorite (NaClO), UV and peracetic acid disinfection treatment of *Salmonella* spp., pathogenic *Campylobacter*, STEC and bacterial indicators in three full-scale municipal wastewater plants. A general reduction in *Salmonella* was observed after disinfection, but these bacteria were detected in one UV-treated sample (culture method) and in 33%, 50% and 17% of samples collected after NaClO, UV and PAA disinfection treatments, respectively (PCR method). A better reduction was also observed under NaClO disinfection for the microbial indicators. Independent of the disinfection treatment, *E. coli* O157:H7 was not detected in the disinfected samples, whereas some samples treated with UV and PAA showed the presence of the *stx1* gene. No reduction in the presence of *stx2* genes was verified for any of the disinfection treatments. *Campylobacter* was not detected in any of the analysed samples. The overall results highlight a better reduction in microbiological parameters with a NaClO disinfection treatment in a full-scale municipal wastewater plant compared with UV and PAA. However, the results indicate that a complete and specific monitoring program is necessary to prevent a possible risk to public health.

## 1. Introduction

Water scarcity is currently one of the main challenges being faced by humans and governments; consequently, water quality protection for drinking purposes, aiming to reduce the impact on human health, represents a priority issue [1]. One of the main sources of water contamination is the release of wastewater that has not been properly treated, representing an important possible contributor to numerous pathogens and chemical contaminants [2,3]. Primary and secondary wastewater treatment typically achieves 90–99.9% enteric microflora reduction, but in some cases, this reduction may be poor. For this reason, an additional conclusive step of effluent disinfection must generally be performed [4,5]. An ideal disinfectant for this step should efficiently remove the pathogenic microorganism’s maximum, avoiding the neoproduction of dangerous and undesirable by-products, and should be inexpensive and technologically compatible [6]. The chlorination is the most widely used method for the disinfection of wastewater, because it showed high effectiveness and low residual effect and cost [7]. Although this treatment inactivates different pathogens, the common application of chlorination in wastewater disinfection has led to concerns regarding the presence of disinfection by-products (such as the total organic halogen) that are cytotoxic, genotoxic and carcinogenic [8]. Thus, alternative disinfection treatments are utilised, such as ultraviolet (UV) radiation, peracetic acid (PAA) and ozonisation. The main physical procedure for the disinfection of wastewater is UV irradiation; one of the advantages of this method is that it produces nontoxic by-products [9]. However, small doses might not be sufficient to inactivate some microorganisms, and they might reverse the radiation effect through photoreactivation or dark repair. In addition, the total suspended solids and turbidity in the sewage were found to cause a lower microbiological reduction. [3,10]. PAA is a strong oxidant that represents different advantages: first, it has a large spectrum of antimicrobial activity, which is not influenced by different values of organic matter, without producing toxic and/or mutagenic by-products; moreover, it does not require dechlorination and it presents a low dependence on pH [11,12]. Although PAA can reduce the main enteric bacteria in wastewater, this disinfectant is associated with microbial regrowth and it shows lower abatement of some microorganisms (e.g., viruses and parasites) [13,14].

The information above on the main techniques applied for wastewater disinfection underlines the need to identify the most suitable processes to treat sewage. In fact, the contamination of surface waters by pathogenic microorganisms, as a consequence of inadequate wastewater disinfection, may promote waterborne disease spread, including those caused by pathogenic *Campylobacter*, Shiga toxin (Stx)-producing *Escherichia coli* (STEC) and *Salmonella* [15,16,17]. Although the predominant mode of *Campylobacter* and STEC transmission to humans is via the consumption of contaminated foods, water can also be a source of human exposure [18,19]. The largest reported waterborne outbreak of *Escherichia coli* O157:H7 in the United States was a consequence of a coinfection with *Campylobacter jejuni*, affecting 775 persons in New York State in August 1999. The epidemiological investigation has identified discharges of wastewater into river as the main source of contamination of drinking water supplies [20]. Although O157:H7 represents the most investigated strain, recent studies also highlight the role of non-O157 STEC strains as pathogens in aquatic ecosystems [21,22]. A large outbreak (1431 cases), ascribed to *Campylobacter* water contamination, was reported in Switzerland in 1998. The outbreak was due to pump failure producing a spill of sewage into the groundwater [23]. Recently, a campylobacteriosis outbreak (39 cases) associated with a municipal water system contaminated by wastewater was reported in Nebraska [24]. Moreover, it is also important to highlight that a possible risk for humans can also derive from the spreading of pathogenic bacteria through irrigation with surface waters contaminated by wastewater effluents. For example, according to the data provided by Centers for Disease Control and Prevention, *Salmonella* is the main aetiological agent involved in the foodborne disease outbreaks (~53.4%) from 2006 to 2017, and the consumption of produce was frequently associated with these outbreaks (~32.7%). The recent literature suggests that irrigation water represent a possible source of *Salmonella* contamination in produce, highlighting its possible role as a transmission vehicle [25].

The aim of this study was to evaluate the reduction obtained with sodium hypochlorite, UV and peracetic acid disinfection treatment of the most important zoonotic bacterial pathogens (*Salmonella* spp., pathogenic *Campylobacter*, and STEC) and typical bacterial indicators of faecal contamination in three different full-scale municipal wastewater plants.

## 2. Results and Discussion

### 2.1. Bacterial Indicators

The presence of *E. coli*, coliform bacteria, enterococci, and *C. perfringens* spores was investigated, comparing the reduction obtained with the three different disinfection treatments (WWTP1 with NaClO, WWTP2 with UV, WWTP3 with PAA). Moreover, the relationship between bacterial concentrations and pathogens was evaluated. The mean concentrations of the faecal indicators found in the three WWTPs are reported in Table S1 (Supplementary Material).

Generally, in the non-disinfected effluents (E), the concentrations of the indicators were higher in WWTP1 and WWTP2 than in WWTP3 (*p* > 0.05). Moreover, in all WWTPs, the bacterial levels observed in non-disinfected effluent were similar to those reported in other studies [3,26,27]. Furthermore, the decreasing trend of the mean concentrations of the four indicators in the three WWTPs was total coliforms > *E. coli* > Enterococci > *C. perfringens*. The mean concentrations of coliform bacteria and *C. perfringens* spores were similar in the non-disinfected effluent of the three WWTPs (ANOVA *p* > 0.05) (Table S2, Supplementary Material). A difference between the mean concentration of *E. coli* and enterococci in the non-disinfected effluents of WWTP1 and WWTP2 with respect to WWTP3 was observed, but this difference was statistically significant only between WWTP1 and WWTP3 (ANOVA *p* < 0.005 for *E. coli* and *p* < 0.05 for enterococci) (Table S3, Supplementary Material).

Moreover, ANOVA highlighted that the bacterial load in the WWTPs was similar between the seasons (*p* > 0.05).

The decreasing trend of the mean concentration of the four indicators in the disinfected effluents (DE) of the three WWTPs was *C. perfringens* ≥ Enterococci > total coliforms > *E. coli* (Table S3, Supplementary Material). This trend was attributable to the different sensitivities of the four indicators to the disinfection treatments applied, as reported by other authors [27,28,29]. The concentrations of the four indicators were lower in WWTP1 than in WWTP2 and WWTP3. The ANOVA and post-hoc tests highlight a significant difference between the mean concentration of total coliforms, *E. coli* and enterococci in the disinfected effluents of WWTP1 vs. WWTP2 and WWTP3. Otherwise, the mean concentrations of *C. perfringens* spores were similar in the disinfected effluent of the three WWTPs (ANOVA *p* > 0.05).

Moreover, a significant difference in the concentrations of the four indicators between the E and DE of each WWTP was observed, with the exception of *C. perfringens* spores in the WWTP3. Therefore, all three disinfection treatments were useful in reducing the microbial concentration in the final effluent (Table S2).

**Figure 1:**
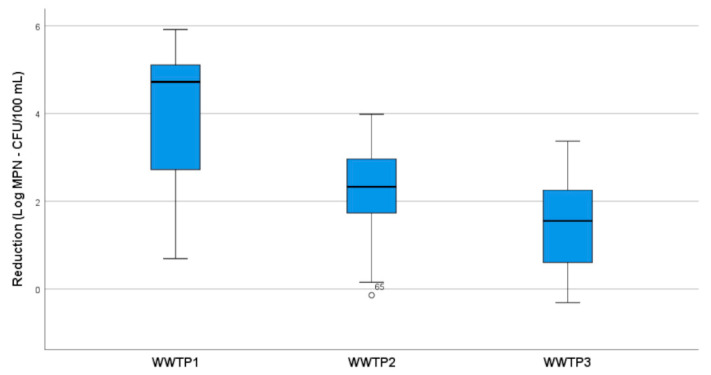
Box plot of microbial reduction by the three WWTPs (WWTP1: NaClO; WWTP2: UV; WWTP3: PAA).

Comparing the total mean reduction in all the indicators for each WWTP (Figure 1), a significant difference was observed among the three WWTPs (ANOVA *p* < 0.0005), particularly between WWTP1 and WWTP2 (post hoc *p* < 0.0005) and between WWTP1 and WWTP3 (post hoc *p* < 0.0005).

**Figure 2:**
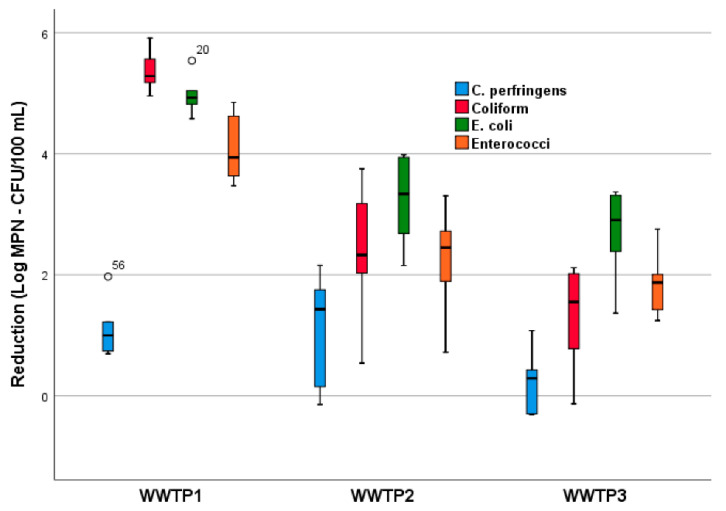
Box plot of reduction of the four indicators by the three WWTPs. (WWTP1: NaClO; WWTP2: UV; WWTP3: PAA).

The mean removal of each microbial indicator by the three WWTPs is shown in Figure 2: a lower reduction was found in the three WWTPs for *C. perfringens* spores, without any significant differences among the three disinfection treatments; otherwise, *E. coli*, coliform bacteria and enterococci reached a high abatement in all plants with significant differences related to the application of the different treatments (NaClO, UV and PAA) (ANOVA *p* < 0.0005).

In particular, the reduction in coliforms, *E. coli* and enterococci in WWTP1 because of NaClO disinfection was significantly greater than those reached in WWTP2 (post hoc *p* < 0.0005, *p* < 0.005 and *p* < 0.005, respectively) and WWTP3 (post hoc *p* < 0.0005, *p* < 0.0005 and *p* < 0.0005, respectively), in which disinfection was made with UV and PAA, respectively. Therefore, NaClO disinfection is confirmed as the most effective treatment in terms of indicator microorganism reduction. It is important to emphasize that all three disinfection processes allow values below the regulatory reference limit for *E. coli* concentration (5 × 10^3^ CFU/100 mL) to be reached with reference to wastewater discharge into surface waters in Italy [30]. However, the non-disinfected effluents of all plants always presented concentrations above this limit, underlining the need for a disinfection step to reduce the microbial impact of wastewater effluents on the receiving surface water. Considering that Italian legislation for the reuse of wastewater effluents establishes a legal limit of 1 Log CFU/100 mL for *E. coli* concentration [31], disinfection with NaClO always reached this goal, except in July, which was probably due to the particularly high concentration of *E. coli* in the non-disinfected effluent. In contrast, the effluents disinfected with UV and PAA did not comply with the limit, except in two samples disinfected by UV (January and July) and in one sample disinfected by PAA (January).

Pearson’s correlation analysis shows a positive correlation, even if not significant, between the dosage of NaClO applied in the WWTP1 and the reduction in coliforms (r = 0.771), of *E. coli* (r = 0.756) and the total mean abatement (r = 0.626). Additionally, for NaClO contact times, positive but not significant correlations were found, with *E. coli* (r = 0.761), *C. perfringens* (r = 0.608) and the total mean abatement (r = 0.631), while a positive and significant correlation with coliform bacteria was found (r = 0.898; *p* < 0.05). These results confirm the sensitivity of coliforms and *E. coli* to NaClO and show that the reduction in *C. perfringens* was mainly dependent on the contact time rather than on the dosage used.

No correlation (Pearson) was found with the dosage and contact time of PAA applied in the WWTP3. No relationship between the parameters of UV treatment and the microbial indicators can be determined because the power of UV lamps and the contact time are fixed.

### 2.2. Pathogenic Bacteria

The results of the detection of *Salmonella* spp., *E. coli* O157:H7 and *Campylobacter* carried out in the three full-scale municipal wastewater plants are reported in Table 1.

*Salmonella* spp. were observed in all the analysed effluents using molecular methods, except for the samples collected in WWTP3 in January 2018, while they were seen in 66.6% of the non-disinfected effluents (E) with the culture method. Analogous percentages of contamination were also reported in other studies [28,32]. The presence of *Salmonella* in the effluents before disinfection treatment underlines that conventional municipal wastewater treatments are not able to remove these bacteria. This finding underlines the importance of using tertiary disinfection treatment to avoid the spread of enteric pathogens in the environment and to prevent a possible risk to public health [4]. Considering the different disinfection treatments, a generally consistent reduction in *Salmonella* contamination was observed; in particular, with the culture-based method, *Salmonella* was detected in a sole sample of the WWTP2 (May 2017), whereas with the PCR method, *Salmonella* was detected in 33% of samples (2/6) collected after NaClO disinfection, in 50% (3/6) after UV treatment and in 17% (1/6) after PAA disinfection. These results highlight that none of the disinfection treatments was able to remove *Salmonella* contamination completely in the final effluent; however, a better reduction was observed with PAA and NaClO disinfection with respect to UV. The same trend was also observed for the microbial indicators. The efficacy of NaClO and PAA in reducing *Salmonella* contamination has been reported in other studies [33,34], where the performances of different WWTP disinfection technologies were evaluated. In line with our results, Veschetti and collaborators [32] reported a similar bactericidal power of PAA and NaClO against *Salmonella* and other microorganisms. The lower disinfection of UV treatment with respect to PAA and NaClO could be due to the photoreactivation mechanism of *Salmonella* and other pathogenic bacteria. In fact, the potential regrowth and repair of pathogenic bacteria (photoreactivation) in UV-disinfected wastewater was reported as a drawback of the real application of this process [10].

Comparing the results obtained with the molecular and culture methods for *Salmonella* detection, a lower contamination was observed in the effluent before disinfection as well as after the treatments using the culture method. These results could be related to the difficulty to isolate the strain from XLD agar because of the presence of interfering microflora (e.g., *Proteus mirabilis*). This methodological problem should be taken into account when the microbiological risk associated with the reuse of wastewater effluent is estimated, considering that Italian regulation prescribes *Salmonella* spp. absence evaluated by the culture method [31].

The results of the PCR analyses showed that 50% (3/6) of the WWTP1 and WWTP3 effluents were positive for *E. coli* O157:H7, while only one of the WWTP2 non-disinfected samples (17%) was contaminated with this pathogenic microorganism. Independent of the disinfection treatment applied, *E. coli* O157:H7 was not detected in any of the examined disinfected samples, demonstrating the effectiveness of removal of this microorganism by the tertiary disinfection treatments investigated.

A total of four (4/6 or 67%), four (4/6 or 67%) and five (5/6 or 83%) effluents before disinfection showed the presence of amplicons corresponding to the *stx1* gene (WWTP1, WWTP2 and WWTP3, respectively). The *stx2* gene was detected only in one effluent (E) in WWTP2 (January 2018). The presence of amplicons corresponding to the *stx1* and *stx2* genes in effluents before disinfection was always associated with the O157:H7 serotype or other STECs, as reported in Table 1. After disinfection, in WWTP1, no samples revealed the presence of the *stx1* amplicon, while WWTP2 and WWTP3 showed two (33%, 2/6) and one (17%, 1/6) sample with the *stx1* gene, respectively. No reduction in the presence of *stx2* genes was verified for the disinfection treatments; in fact, two (2/6 or 33%), one (1/6 or 17%) and one (1/6 or 17%) effluent after disinfection revealed the presence of amplicons corresponding to the *stx2* gene in WWTP1, WWTP2 and WWTP3, respectively. As reported in Table 2, three samples (DE WWTP1 and WWTP3 September 2017 and DE WWTP1 May 2018) tested positive for *stx* genes, but they were not associated with the presence of the STEC serogroups investigated in this study, in agreement with the results obtained in our previous studies [28,35]. This finding was probably related to the presence of bacteriophages carrying *stx1*/*stx2* genes, since their role in the dissemination of such sequences among STEC and *Shigella* strains and among other waterborne bacteria is well known [36,37]. Another reason for the abovementioned *stx1*/*stx2* amplification products could be the presence of STEC serogroups other than those targeted in this research.

It is also important to consider that the presence of *stx* genes is essential to infection; however, other virulence factors (e.g., the *eae* gene) could be involved [38]. In our study, the *eae* gene (coding for the virulence factor intimin) was revealed in only one sample of disinfected effluent, but in this sample the *stx1*/*stx2* genes were absent.

*Campylobacter* were not detected in any of the samples analysed because no amplicons corresponding to genus-specific 16S rRNA (*Campylobacter* spp.) and species-specific mapA and ceuE genes (for *C. jejuni* and *C. coli* species) were detected. Considering the absence of contamination by pathogenic *Campylobacter*, no conclusions about the reduction in the different disinfection treatments can be drawn.

To verify the correlation between the presence of emerging pathogens and the counts of faecal indicators such as *E. coli*, coliforms, enterococci and *C. perfringens* spores, a logistic binary regression analysis was performed. No associations between the presence of O157:H7 genes, the *eae* gene (intimin), *stx1* gene (Shiga-like toxin I), *stx2* gene (Shiga-like toxin II), invA gene (*Salmonella* spp.) and the counts of microbiological indicators (*E. coli*, enterococci, *C. perfringens* spores and coliforms) were observed (*p* > 0.05). Moreover, no relationships were observed between *Salmonella* spp. contamination revealed by the culture method and faecal indicators or other pathogens (*p* > 0.05). These results suggest that the common bacterial indicators of faecal contamination in municipal wastewater samples seem to not be reliable indicators of pathogenic bacteria presence. These considerations are in agreement with other studies on relationship between bacterial indicators and pathogenic bacteria, including *Salmonella* spp. and STEC [39,40].

To conclude, the overall results obtained underline a better reduction in microbiological parameters monitored using sodium hypochlorite as a disinfection treatment in a full-scale municipal wastewater plant with respect to UV and PAA. However, pathogen detection with molecular methods revealed the presence of *Salmonella* contamination and *stx2* genes in effluents disinfected with NaClO. This finding highlights the need for a complete and specific monitoring program to prevent possible risks to public health, also considering the lack of correlation between pathogens and microbial indicators.

## 3. Materials and Methods

### 3.1. Bacterial Strains and Culture Media

*C. jejuni* (ATCC 33291), *E. coli* O157:H7 (NCTC 129, nontoxigenic strain encoding the *eae* gene), and *S. typhimurium* (ATCC 14028) were used as reference strains. The bacteria utilized in this study were cultivated as reported in [28,35].

### 3.2. Sampling

Effluents before and after disinfection treatment were collected during six sampling periods (September 2017, November 2017, January 2018, March 2018, May 2018 and July 2018) from three Italian wastewater treatment plants (WWTP1—population equivalent of 34000, WWTP2—population equivalent of 8000, and WWTP3—population equivalent of 8000). The different wastewater treatment plants are characterized by a disinfection step with NaClO (15% *w/w*), UV lamp and PAA (15% *w/w*) for WWTP1, WWTP2 and WWTP3, respectively. Chemical–physical characteristics of non-disinfected wastewater are reported in Table 3, and information about disinfection conditions in the sampling period is reported in Table 4. After the sampling, wastewater samples were maintained at +4 °C and analyzed within 24 h.

### 3.3. Microbiological Analyses for Pathogen Detection

Effluents before and after disinfection were tested for pathogen detection. During each sampling, a raw sewage sample spiked with a high concentration of pathogens (~10^6^ CFU/100 mL) was used as a positive control. The concentration, enrichment, DNA extraction and PCR/real-time PCR steps were carried out as reported in our previous studies [35,41,42]. *Salmonella* spp. detection was also evaluated using the culture method. Briefly, samples were pre-enriched in peptone water (Oxoid), enriched in selective media (Rappaport Vassiliadis Broth, RV, Oxoid) and streaked on specific media (Xylose Lysine Deoxycholate agar Oxoid) [43]. The identification of selected bacterial colonies was carried out using an API^®^ 20E kit (BioMerieux, Marcy L’Etoile, France).

### 3.4. Microbiological Analyses for the Detection of Microbial Indicators

*E. coli*, enterococci, *Clostridium perfringens* spores and coliforms were analysed in all samples. In brief, the membrane filtration method was used to process wastewater samples for *C. perfringens* enumeration, as reported by ISO 14189:2013 [44]. Wastewater samples were assayed for *E. coli*, coliforms, and enterococci with a commercial semiautomated quantification method (Quanti-TrayTM 2000, IDEXX Laboratories, Milan, Italy). With Quanti-Tray, a 100 mL sample was added to a specific substrate for each microorganism, and the mixture was divided into 51 wells and incubated at a specific temperature. Then the standard method, based on the Most Probable Number (MPN) approach, was used to determine the number of bacteria in the original sample. The Colilert-18 test uses a growth substrate with specific indicators (ONPG and MUG) to detect coliforms, and *E. coli*. Coliforms use their β-galactosidase enzyme to metabolize ONPG and change it from colorless to yellow. *E. coli* use β-glucuronidase to metabolize MUG and create fluorescence. The Enterolert Test uses another growth substrate to detect enterococci. When enterococci utilize their ß-glucosidase enzyme to metabolize Enterolert’s nutrient-indicator (4-methyl-umbelliferyl ß-D-glucoside) the sample fluoresces, the number of positive wells are counted and referred to the MPN table provided to obtain the Most Probable Number of bacteria (MPN/100 mL of sample). This method has been approved by U.S. EPA and it has been included in Standard Methods for Examination of Water and Wastewater.

### 3.5. Statistical Analyses

IBM SPSS Statistics version 26.0 (IBM, Segrate, Italy) for Windows was used for statistical analyses. The relationship between the indicator bacteria load (log 10) and the presence/absence of pathogens was carried out with binary logistic regression. The reduction in indicator load using the different disinfection treatments was analyzed with ANOVA and Tukey’s post-hoc tests. The association between the disinfection conditions (quantity and contact time) and the reduction in indicators was performed using Pearson’s correlation.

## Figures and Tables

**Table 1:** Detection of *Salmonella* spp., *E. coli* O157:H7, *E. coli* virulence genes and *Campylobacter* (spp., *C. coli*, and *C. jejuni*) in the untreated and disinfected samples.

Sample	Sampling Month	WWTP	*Salmonella*	*E. coli* O157:H7					*Campylobacter*			*Salmonella*
			invA	O157	H7	Intimin	SLT-I	SLT-II	Genus	*C. jejuni*	*C. coli*	Culture Method
E	September 2017	1	+	+	+	−	+	−	−	−	−	+
DE	September 2017	1	−	−	−	−	−	+	−	−	−	−
E	November 2017	1	+	+	+	−	+	−	−	−	−	− *
DE	November 2017	1	+	−	+	−	−	−	−	−	−	−
E	January 2018	1	+	+	+	−	−	−	−	−	−	+
DE	January 2018	1	−	−	+	−	−	−	−	−	−	−
E	March 2018	1	+	−	+	−	+	−	−	−	−	+
DE	March 2018	1	−	−	−	+	−	−	−	−	−	−
E	May 2018	1	+	−	−	−	+	−	−	−	−	+
DE	May 2018	1	−	−	−	−	−	+	−	−	−	−
E	July 2018	1	+	−	−	−	−	−	−	−	−	+
DE	July 2018	1	+	−	−	−	−	−	−	−	−	−
E	September 2017	2	+	+	+	−	+	−	−	−	−	+
DE	September 2017	2	−	−	+	−	+	+	−	−	−	−
E	November 2017	2	+	−	+	+	+	−	−	−	−	− *
DE	November 2017	2	−	−	+	−	−	−	−	−	−	−
E	January 2018	2	+	−	+	−	−	+	−	−	−	− *
DE	January 2018	2	−	−	−	−	−	−	−	−	−	−
E	March 2018	2	+	−	+	−	+	−	−	−	−	+
DE	March 2018	2	+	−	+	−	−	−	−	−	−	−
E	May 2018	2	+	−	−	−	+	−	−	−	−	+
DE	May 2018	2	+	−	−	−	+	−	−	−	−	+
E	July 2018	2	+	−	−	−	−	−	−	−	−	+
DE	July 2018	2	+	−	−	−	−	−	−	−	−	−
E	September 2017	3	+	+	+	−	+	−	−	−	−	+
DE	September 2017	3	−	−	−	−	−	+	−	−	−	−
E	November 2017	3	+	−	+	−	+	−	−	−	−	− *
DE	November 2017	3	−	+	−	−	−	−	−	−	−	−
E	January 2018	3	−	−	+	−	+	−	−	−	−	− *
DE	January 2018	3	−	+	+	−	+	−	−	−	−	−
E	March 2018	3	+	+	+	−	+	−	−	−	−	− *
DE	March 2018	3	−	−	+	−	−	−	−	−	−	−
E	May 2018	3	+	−	−	−	+	−	−	−	−	+
DE	May 2018	3	−	−	−	−	−	−	−	−	−	−
E	July 2018	3	+	−	−	−	−	−	−	−	−	+
DE	July 2018	3	+	−	−	−	−	−	−	−	−	−

E: Effluent; DE: Disinfected Effluent; +: positive; −: negative; WWTP: Wastewater Treatment Plant; WWTP1: NaClO; WWTP2: UV; WWTP3: PAA; * *Salmonella* spp. probably present but not identified for the presence of *Proteus mirabilis*.

**Table 2:** Detection of non-O157 STECs by real-time PCR in untreated and disinfected samples.

Sample	Sampling Month	WWTP	*E. coli* Serogroup							
			O157	O103	O26	O145	O111	O104	SLT-I	SLT-II
DE	September 2017	1	−	−	−	−	−	−	−	+
E	March 2018	1	−	+	+	−	+	+	+	−
E	May 2018	1	−	+	+	+	+	+	+	−
DE	May 2018	1	−	−	−	−	−	−	−	+
E	November 2017	2	−	+	+	−	+	+	+	−
E	January 2018	2	−	+	+	−	+	+	−	+
E	March 2018	2	−	+	+	+	+	+	+	−
E	May 2018	2	−	+	+	+	+	+	+	−
DE	May 2018	2	−	+	+	+	+	+	+	−
DE	September 2017	3	−	−	−	−	−	−	−	+
E	November 2017	3	−	+	−	−	+	+	+	−
E	January 2018	3	−	+	+	+	+	+	+	−
E	May 2018	3	−	+	+	+	+	+	+	−

E: Effluent; DE: Disinfected Effluent; +: positive; −: negative; WWTP: Wastewater Treatment Plant; WWTP1: NaClO; WWTP2: UV; WWTP3: PAA.

**Table 3:** Chemical–physical characteristics (mean ± SD) of the non-disinfected effluent in the three WWTPs.

WWTP	TSS (mg/L)	COD (mg/L)	pH
1	11.04 ± 4.53	28.38 ± 8.86	6.55 ± 0.57
2	11.22 ± 5.53	25.32 ± 9.02	6.86 ± 0.32
3	10.08 ± 5.77	21.38 ± 7.93	6.90 ± 0.31

WWTP: Wastewater Treatment Plant; TSS: Total Solid Suspended; COD: Chemical Oxygen Demand.

**Table 4:** Disinfection conditions in the different wastewater treatment plants monitored.

WWTP	Sampling Period	Flow (m^3^/die)	Medium Flow (m^3^/h)	Contact Time (min)	Disinfectant Agent	Quantity * (mg/L) or (mJ/cm^2^)
1	September 2017	16,431	685	36.81	NaClO	0.69
1	November 2017	11,720	488	51.60	NaClO	1.50
1	January 2018	13,088	545	46.21	NaClO	1.34
1	March 2018	14,126	589	42.81	NaClO	1.24
1	May 2018	15,448	644	39.15	NaClO	0.73
1	July 2018	17,774	741	34.03	NaClO	0.64
2	September 2017	2451	102	/	UV	54
2	November 2017	1605	67	/	UV	54
2	January 2018	1638	68	/	UV	54
2	March 2018	1394	58	/	UV	54
2	May 2018	2851	119	/	UV	54
2	July 2018	1485	62	/	UV	54
3	September 2017	1556	65	18.66	PAA	3.93
3	November 2017	1710	71	16.98	PAA	3.58
3	January 2018	1528	64	19.01	PAA	4.01
3	March 2018	1920	80	15.12	PAA	3.19
3	May 2018	2436	102	11.92	PAA	2.51
3	July 2018	2218	92	13.09	PAA	2.76

* mg/L: concentration of disinfectant (NaClO or PAA); mJ/cm^2^: dosage UV.

## Data Availability

All data presented in this study are available in this manuscript and Supplementary Materials.

## Supplementary Materials

The following are available online at https://www.mdpi.com/2076-0817/10/2/182/s1, Table S1: Faecal indicators in the untreated (E) and disinfected effluents (DE). Table S2: Results of the ANOVA and post-hoc Tukey test related to the four indicators in each WWTP. Table S3: Results of the ANOVA and post-hoc Tukey test related to each indicator.
