# Supplementary material for: Comparison of UV, Peracetic Acid and Sodium Hypochlorite Treatment in the Disinfection of Urban Wastewater
Source: Pathogens. 2021 Feb 9;10(2):182. doi: 10.3390/pathogens10020182 (PMC7914577; doi:10.3390/pathogens10020182)
Supplement: Supplementary file 1 [file pathogens-10-00182-s001.pdf]

Table S1. Faecal indicators in the untreated (E) and disinfected effluents (DE).

Sample	Sampling month	WWTP	Coliform	*E. coli*	Enterococci	*C. perfringens* spore	Regulatory limit (*E.coli*)*
			Log MPN/100 ml			Log CFU/100 ml	
E	September 2017	1	5,64 ± 0,14	4,81 ± 0,11	4,12 ± 0,08	3,26 ± 0,11	3,7
DE	September 2017	1	0,30 ± 0,02	< LOD	0,49 ± 0,02	2,35 ± 0,04	3,7
E	November 2017	1	5,91 ± 0,07	5,54 ± 0,10	4,79 ± 0,13	3,15 ± 0,19	3,7
DE	November 2017	1	< LOD	< LOD	0,18 ± 0,21	1,18 ± 0,04	3,7
E	January 2018	1	5,56 ± 0,16	5,04 ± 0,01	4,44 ± 0,08	4,15 ± 0,06	3,7
DE	January 2018	1	< LOD	< LOD	0,72 ± 0,01	3,41 ± 0,29	3,7
E	March 2018	1	5,17 ± 0,08	4,91 ± 0,01	4,15 ± 0,10	3,36 ± 0,27	3,7
DE	March 2018	1	< LOD	< LOD	< LOD	2,15 ± 0,10	3,7
E	May 2018	1	5,22 ± 0,04	4,58 ± 0,11	3,95 ± 0,12	3,65 ± 0,36	3,7
DE	May 2018	1	< LOD	< LOD	0,48 ± 0,01	2,95 ± 0,24	3,7
E	July 2018	1	6.79 ± 0,26	6,16 ± 0,22	5,34 ± 0,09	4,07 ± 0,52	3,7
DE	July 2018	1	1,83 ± 0,40	1,22 ± 0,84	0,49 ± 0,01	2,99 ± 0,18	3,7
E	September 2017	2	5,31 ± 0,11	4,67 ± 0,10	4,03 ± 0,05	3,97 ± 0,01	3,7

DE	September 2017	2	3,28 ± 0,14	2,52 ± 0,07	1,61 ± 0,09	3,82 ±0,07	3,7
E	November 2017	2	5,39 ± 0,07	5,22 ± 0,12	4,22 ± 0,17	3,10 ±0,09	3,7
DE	November 2017	2	2,22 ± 0,19	1,28 ± 0,43	2,32 ± 0,14	1,95 ± 0,03	3,7
E	January 2018	2	5,27 ± 0,03	4,92 ± 0,04	4,10 ± 0,03	4,69 ± 0,14	3,7
DE	January 2018	2	1,52 ± 0,26	0,93 ±0,01	0,80 ± 0,01	2,53 ± 0,01	3,7
E	March 2018	2	4,97 ± 0,01	4,28 ± 0,07	3,82 ± 0,11	3,75 ± 0,01	3,7
DE	March 2018	2	2,62 ± 0,20	1,53 ± 0,05	1,10 ± 0,41	2,00 ± 0,53	3,7
E	May 2018	2	5,45 ± 0,08	4,94 ± 0,08	4,24 ± 0,06	3,50 ± 0,68	3,7
DE	May 2018	2	3,14 ± 0,05	2,26 ± 0,08	1,77 ± 0,19	3,64 ± 0,05	3,7
E	July 2018	2	5,35 ± 0,18	4,64 ± 0,16	3,53 ± 0,06	3,58 ± 0,20	3,7
DE	July 2018	2	4,81 ± 0,14	0,71 ± 0,04	2,81 ± 0,13	1,87 ± 0,30	3,7
E	September 2017	3	4,66 ± 0,08	3,92 ± 0,05	3,59 ± 0,12	3,78 ± 0,02	3,7
DE	September 2017	3	3,88 ± 0,08	1,27 ± 0,49	2,16 ± 0,08	4,08 ± 0,06	3,7
E	November 2017	3	4,14 ± 0,07	3,47 ± 0,18	3,56 ± 0,08	3,06 ± 0,06	3,7
DE	November 2017	3	2,55 ± 0,18	1,09 ± 0,01	2,32 ± 1,14	2,76 ± 0,27	3,7

E	January 2018	3	4,41 ± 0,05	3,89 ± 0,10	3,57 ± 0,42	4,03 ± 0,27	3,7
DE	January 2018	3	2,39 ± 0,04	0,74 ± 0,71	1,57 ± 0,02	2,95 ± 0,04	3,7
E	March 2018	3	5,02 ± 0,23	4,39 ± 0,11	3,84 ± 0,10	3,52 ± 0,04	3,7
DE	March 2018	3	3,51 ± 0,29	1,08 ± 0,02	1,09 ± 0,12	3,25 ± 0,12	3,7
E	May 2018	3	4,92 ± 0,02	4,41 ± 0,04	3,48 ± 0,15	3,48 ± 0,10	3,7
DE	May 2018	3	2,81 ± 0,09	1,04 ± 0,03	1,51 ± 0,03	3,79 ± 0,04	3,7
E	July 2018	3	4,16 ± 0,10	3,23 ± 0,18	2,97 ± 0,10	3,60 ± 0,17	3,7
DE	July 2018	3	4,29 ± 0,14	1,86 ± 0,29	1,19 ± 0,23	3,18 ± 0,21	3,7

WWTP1: NaClO; WWTP2: UV; WWTP3: PAA; LOD, limit of detection, *: regulatory reference limit for *E. coli* concentration ($5 X 10^3$ CFU/100 ml) with reference to wastewater discharge into surface waters in Italy [30]

Table S2. Results of the ANOVA and post-hoc Tukey test related to the four indicators in each WWTP.

**Plant**	**Sampling point**	**ANOVA p**	***VS***	**Post hoc p**
**WWTP1**	E1	0.0005	Coliform vs Enterococci	0.005
			Coliform vs *C. perfringens*	0.0005
			E.coli vs *C. perfringens*	0.0005
	E1D	0.0005	Coliform vs *C. perfringens*	0.0005
			E.coli vs *C. perfringens*	0.0005
			Enterococci vs *C. perfringens*	0.0005
	REDUCTION E1 – E1D	0.0005	Coliform vs Enterococci	0.0005
			Coliform vs *C. perfringens*	0.0005
			E.coli vs Enterococci	0.005
			E.coli vs *C. perfringens*	0.0005
			Enterococci vs *C. perfringens*	0.0005

**WWPT2**	E2	0.0005	Coliform vs Enterococci	0.0005
			Coliform vs *C. perfringens*	0.0005
			E.coli vs Enterococci	0.005
			E.coli vs *C. perfringens*	0.0005
	REDUCTION E2 –E2D	0.01	*E.coli* vs *C. perfringens*	0.005
**WWTP3**	E3	0.0005	Coliform vs *E. coli*	0.05
			Coliform vs Enterococci	0.0005
			Coliform vs *C. perfringens*	0.001
	E3D	0.0005	Coliform vs *E. coli*	0.0005
			Coliform vs Enterococci	0.0005
			E.coli vs *C. perfringens*	0.0005
			Enterococci vs *C. perfringens*	0.0005
	REDUCTION E3 – E3D	0.0005	Coliform vs *E. coli*	0.05
			E.coli vs *C. perfringens*	0.0005
			Enterococci vs *C. perfringens*	0.005

Table S3. Results of the ANOVA and post-hoc Tukey test related to each indicator

**Indicator**	**ANOVA p**	**VS**	**Post hoc p**
**Total coliform**	0.0005	E1 vs E1D	0.0005
		E2 vs E2D	0.0005
		E3 vs E3D	0.05
		E1D vs E2D	0.0005
		E1D vs E3D	0.0005
E. coli	0.0005	E1 vs E1D	0.0005
		E1 vs E3	0.005
		E2 vs E2D	0.0005

		E3 vs E3D	0.0005
		E1D vs E2D	0.005
		E1D vs E3D	0.05
**Enterococci**	0.0005	E1 vs E1D	0.0005
		E1 vs E3	0.05
		E3 vs E3D	0.0005
		E3 vs E3D	0.0005
		E1D vs E2D	0.0005
		E1D vs E3D	0.005
C. perfringens	0.005	E1 vs E1D	0.05
		E2 vs E2D	0.05
